# On the Elimination of Fast Variables from the Langevin Equation

**DOI:** 10.3390/e26100821

**Published:** 2024-09-26

**Authors:** Dick Bedeaux

**Affiliations:** PoreLab, Department of Chemistry, Norwegian University of Science and Technology, NO-7491 Trondheim, Norway; dick.bedeaux@ntnu.no

**Keywords:** Langevin equation, fluctuation–dissipation theorems, fast and slow variables, Onsager symmetry relations, correlation functions

## Abstract

In a multivariable system, there are usually a number of relaxation times. When some of the relaxation times are shorter than others, the corresponding variables will decay to their equilibrium value faster than the others. After the fast variables have decayed, the system can be described with a smaller number of variables. From the theory of nonequilibrium thermodynamics, as formulated by Onsager, we know that the coefficients in the linear flux–force relations satisfy the so-called Onsager symmetry relations. The question we will address in this paper is how to eliminate the fast variables in such a way that the coefficients in the reduced description for the slow variables still satisfy the Onsager relations. As the proof that Onsager gave of the symmetry relations does not depend on the choice of the variables, it is equally valid for the subset of slow variables. Elimination procedures that lead to symmetry breaking are possible, but do not consider systems that satisfy the laws of nonequilibrium thermodynamics.

## 1. Introduction

In 1931, Onsager formulated [1,2,3,4] the classical theory of nonequilibrium thermodynamics. He showed that the coefficients in the linear flux–force relations for a multivariable system satisfy the so-called Onsager symmetry relations. When one describes the system with the Langevin equation, one adds random fluxes, the averages of which are zero. The correlation functions of the random fluxes are given by fluctuation–dissipation theorems [5,6,7]. These correlation functions are also symmetric for the interchange of the random fluxes.

When some of the variables decay to their equilibrium value faster than the others, one may after this decay describe the system with a smaller number of slow variables. An example of this is a multi-component fluid. The pressure distribution is the first to reach equilibrium. After this, the fluid can be treated as incompressible. Subsequently, the relaxation of the temperature is usually faster than the diffusion of the components. The advantage is that one may simplify the description to a smaller number of variables on longer time scales.

There are many ways in which one may eliminate fast variables. The proper methodology depends on the particular system considered. Some authors [8,9,10,11] claim that in the reduced description, the symmetry relations are no longer valid. For the systems they consider, and with their methodology to reduce the description, this is indeed what they find. Van Kampen [9] gives a very clear explanation of how to eliminate fast variables. In the systems, he considers that it is possible to identify a small parameter. The solution is then expanded in this parameter. The reduced description may no longer satisfy Onsager symmetry [8,9].

The unfortunate (or fortunate) fact is that the proof that Onsager gave of the symmetry relations does not depend on the choice of the variables, and it is equally valid for the subset of slow variables as for the full set. It is therefore clear that the systems some authors [8,9,10,11] consider, after eliminating the fast variables, no longer belong to the well established field of nonequilibrium thermodynamics. It is known that in the non-linear case the symmetry relations are no longer valid [12], but that is not the issue now.

The question addressed in this paper is how to reduce the description to slow variables such that the Onsager coefficients and the corresponding correlation functions still satisfy the symmetry relations. In the Section 2, I discuss the Langevin equation for *n* variables. It contains a n×n matrix of linear coefficients and *n* thermal random forces. The distribution of the variables around their equilibrium value is given. The average of the random forces is zero. Their auto-correlation functions are given by fluctuation–dissipation theorems in terms of the symmetric Onsager coefficients. In the Section 3, I discuss how to distinguish the fast and the slow variables. The equilibrium distribution of the slow variables is discussed in the Section 4. The derivation of the Langevin equation for the slow variables, including the verification of the symmetry of the Onsager coefficients and the correlation functions, is given in Section 5. The choice of physical variables is shortly discussed in Section 6, and this ends with a conclusion.

## 2. The Langevin Equation

Consider a system described by the variables $a=(a_{1},...,a_{n})$. These variables are chosen such that their equilibrium value is equal to zero, $a_{eq}=0$. I restrict myself to the case that the variables are either all symmetric or all asymmetric for time reversal. Given the entropy production, one has a set of conjugate fluxes and forces. Choosing all variables odd or even is equivalent to using a mixed set. As such, the choice of all even or all odd is no restriction. The velocities of a collection of Brownian particles are a good example of a set of odd variables. The extension to have mixed variables that are both symmetric and asymmetric for time reversal is straightforward [13]. The time dependence is given by the Langevin equation [13]:

(1) $$\frac{da(t)}{dt}=-L.a\left(t\right)+f\left(t\right)$$

Here, L is a n×n matrix of linear coefficients and f is the thermal random force, which is Gaussian and white. The average of the random force is zero

(2) $$\langle f(t)\rangle =0$$

The correlations are given by

(3) $$\langle f\left(t\right)f\left(t^{\prime }\right)\rangle =2𝓁\delta \left(t-t^{\prime }\right)$$

where *ℓ* is the n×n Onsager matrix. Given the Gaussian nature of the random force, one may also calculate the higher order correlation functions. The Onsager relations imply that the matrix *ℓ* is symmetric.

The equilibrium distribution is given by

(4) $$P_{eq}\left(a\right)=\exp\left(S\left(a\right)/k_{B}\right)/\int da^{\prime }\exp\left(S\left(a^{\prime }\right)/k_{B}\right)$$

where S(a) is the entropy and $k_{B}$ is Boltzmann’s constant. Expanding the entropy to second order in a, one has

(5) $$S\left(a\right)=S_{eq}-\frac{1}{2}k_{B}a.g.a$$

where it is used so that the entropy has a maximum in equilibrium. The matrix g is symmetric and is given by

(6) $$g=-\frac{1}{k_{B}}\frac{\partial^{2}}{\partial a^{2}}S\left(a\right)$$

Equation (5) together with Equation (4) gives

(7) $$P_{eq}\left(a\right)={\left(2\pi \right)}^{-n/2}\sqrt{\det g}\exp\left(-\frac{1}{2}a.g.a\right)$$

This gives

(8) $${\langle a\rangle }_{eq}=0$$

and

(9) $${\langle \mathrm{aa}\rangle }_{eq}=g^{-1}$$

Given the Gaussian nature of $P_{eq}$, one may also calculate the higher order correlation functions.

The random force in the Langevin equation must have the appropriate strength in order to generate the proper size of the fluctuations around the equilibrium. This is found to imply that

(10) $$L=𝓁.g\;\;\;\;\mathrm{or}\;\;\;\;𝓁=L.g^{-1}$$

It should be realised that, while both *ℓ* and g are symmetric, L is not necessarily symmetric. One has

(11) $$L^{T}=g.𝓁$$

for the transposed matrix. The superscript *T* indicates the transpose of a matrix.

The solution of the Langevin Equation (1) may be written in the following form

(12) $$a\left(t\right)=\exp\left(-Lt\right).a\left(0\right)+\int_{0}^{t}dt^{\prime }\exp\left(-L\left(t-t^{\prime }\right)\right).f\left(t^{\prime }\right)$$

where a(0) is the value of a at time zero. The average of this equation gives

(13) $$\langle a(t)\rangle =\exp\left(-Lt\right).a\left(0\right)$$

The thermal fluctuations around the average are

(14) $$\delta a\left(t\right)\equiv a\left(t\right)-\langle a(t)\rangle =\int_{0}^{t}dt^{\prime }\exp\left(-L\left(t-t^{\prime }\right)\right).f\left(t^{\prime }\right)$$

The average of this fluctuation is by definition zero. The correlation function is found to be

(15) $$\langle \delta a\left(t\right)\delta a\left(t^{\prime }\right)\rangle =\exp\left(-L\left(t-t^{\prime }\right)\right).g^{-1}=g^{-1}.\exp\left(-L^{T}\left(t-t^{\prime }\right)\right)$$

The second equality follows by expanding the exponents together with Equations (10) and (11). Comparing Equations (13) and (15) one sees that, as is usual, the relaxation of the correlation function and of the average are directly related.

In order to obtain more information about the relaxation times, we calculate the eigenvalues

(16) $$L.e_{\nu }^{r}=\lambda_{\nu }e_{\nu }^{r}\;\;\;\;\mathrm{and}\;\;\;\;e_{\nu }^{l}.L=\lambda_{\nu }e_{\nu }^{l}$$

where $e_{\nu }^{r}$ and $e_{\nu }^{l}$ are the right and the left eigenvectors, respectively. These eigenvectors satisfy the following orthogonality condition:

(17) $$e_{\nu }^{l}.e_{\nu^{\prime }}^{r}=\delta_{\nu \nu^{\prime }}$$

They furthermore satisfy the following completeness relations:

(18) $$\sum_{\nu =1}^{n}e_{\nu }^{r}e_{\nu }^{l}=1\;\;\;\mathrm{and}\;\;\;\sum_{\nu =1}^{n}e_{\nu }^{l}e_{\nu }^{r}=1$$

The two forms of the completeness relation follow on from each other by taking the transpose of the matrix.

## 3. Fast and Slow Variables

For convenience, the eigenvalues are ordered such that Re$\lambda_{1}$ is the smallest one, Re$\lambda_{2}$ is the next smallest one, etc., until Re$\lambda_{n}$, which is the largest one. The real part of the first eigenvalue gives the inverse relaxation time for the slowest decay, while the real part of the last eigenvalue gives the inverse relaxation time for the fastest decay. I want to consider in particular a case where a subset of eigenvalues is much smaller than the rest of the eigenvalues. One may then define the time τ such that

(19) τReλν≪1forν≤mandτReλν≫1forν>m

When t≫τ, the fast decaying modes are equilibrated. Only *m* independent slow modes are then sufficient to describe the system for t≫τ. It is this reduction to a smaller subset of variables which will be studied. Given the validity of Equation (19), one may choose τ=10/Reλm.

For large enough times one has

(20) $$\langle a_{\nu }\left(t\right)\rangle \equiv e_{\nu }^{l}.\langle a(t)\rangle =0\;\;\;\mathrm{for}\;\;\;t\gg \tau \;\;\;\mathrm{and}\;\;\;\nu >m$$

This gives $\left(n-m\right)$ relations between the average variables for t≫τ. For the correlation functions, one similarly finds

(21) $$\begin{array}{ccc} \langle \delta a_{\nu }\left(t\right)\delta a\left(t^{\prime }\right)\rangle & = & \langle \delta a\left(t\right)\delta a_{\nu }\left(t^{\prime }\right)\rangle =\\ e_{\nu }^{l}.\langle \delta a\left(t\right)\delta a\left(t^{\prime }\right)\rangle & = & \langle \delta a\left(t\right)\delta a\left(t^{\prime }\right)\rangle .e_{\nu }^{l}=0\;\;\;\mathrm{for}\;\;\;\left|t-t^{\prime }\right|\gg \tau \;\;\;\mathrm{and}\;\;\;\nu >m \end{array}$$

The equalities follow from the symmetry of the correlation function. Equation (21) implies that for $\left|t-t^{\prime }\right|\gg \tau$, only a m×m submatrix remains for the *m* remaining independent variables.

After the fast modes have decayed, the remaining variables and the corresponding correlation matrix appear to decay from a different initial condition given by

(22) $$\langle a(\tau =0)\rangle =\sum_{\nu =1}^{m}e_{\nu }^{r}e_{\nu }^{l}.a\left(0\right)$$

for the average and

(23) $${\langle \delta a(t)\delta a(t-\tau )\rangle }_{\tau =0}=\sum_{\nu ,\nu^{\prime }=1}^{m}e_{\nu }^{r}e_{\nu }^{l}.g^{-1}.e_{\nu^{\prime }}^{l}e_{\nu^{\prime }}^{r}$$

for the correlation matrix. While the modified initial condition for the average seems rather obvious, it has been considered to be surprising for the correlation function [9,10,11,12,13]. Let me illustrate this by an example. Consider the velocity distribution of a Brownian particle suspended in a fluid. From ensemble theory, it follows that the distribution of the velocity v is given by the Maxwell distribution

(24) $$P_{eq}\left(v\right)={\left(2\pi Mk_{B}T\right)}^{-3/2}\exp\left(-\frac{v^{2}}{2Mk_{B}T}\right)$$

where *M* is the mass of the Brownian particle. It is known that the compression modes of the surrounding fluid are fast. Consider a sound velocity of 1000 m.s^−1^ and a particle size of 1 micron; this defines a time scale of $\tau =10^{-9}$ s. When one measures the velocity of the particle with a time resolution large compared to τ, one sees a particle which carries along a vortex with fluid. On this time scale, it instantaneously adjusts its velocity to the velocity of the particle. The resulting modified initial velocity distribution is then

(25) $$P_{eq}\left(v\right)={\left[2\pi \left(M+\frac{1}{2}M_{fl}\right)k_{B}T\right]}^{-3/2}\exp\left[-\frac{v^{2}}{2\left(M+\frac{1}{2}M_{fl}\right)k_{B}T}\right]$$

where $M_{fl}$ is the mass of the displaced fluid. This result is now generally accepted, but for a while it was controversial [14,15,16,17,18].

## 4. The Reduced Equilibrium Distribution of the Slow Amplitudes

Define the projection on the left eigenvectors by

(26) $$a_{\nu }\left(t\right)\equiv e_{\nu }^{l}.a\left(t\right)\;\;\;\mathrm{for}\;\;\;\nu =1,...,n$$

These amplitudes will be chosen as an alternative set of variables. For t≫τ, only the first *m* of these variables remain. The rest have equilibrated. The reduced equilibrium distribution for the slow variables is given by

(27) $$\begin{array}{ccc} P_{eq}^{slow}(a_{\nu };\nu & = & 1,...,m)\equiv \int \left(\prod_{\nu =m+1}^{n}da_{\nu }\right)P_{eq}\left(a\right)\\ & = & {\left(2\pi \right)}^{-n/2}{\left(\det g\right)}^{1/2}\int \left(\prod_{\nu =m+1}^{n}da_{\nu }\right)\exp\left(-\frac{1}{2}a.g.a\right)\\ & = & {\left(2\pi \right)}^{-n/2}{\left(\det g\right)}^{1/2}\int \left(\prod_{\nu =m+1}^{n}da_{\nu }\right)\exp\left(-\frac{1}{2}\sum_{\nu^{\prime },\nu^{\prime \prime }=1}^{n}a_{\nu^{\prime }}g_{\nu^{\prime }\nu^{\prime \prime }}a_{\nu^{\prime \prime }}\right) \end{array}$$

where

(28) $$g_{\nu^{\prime }\nu^{\prime \prime }}\equiv e_{\nu^{\prime }}^{l}.g.e_{\nu^{\prime \prime }}^{l}$$

From the nature of the integration, it is clear that the reduced equilibrium distribution is again Gaussian

(29) $$P_{eq}^{slow}\left(a_{\nu };\nu =1,...,m\right)={\left(2\pi \right)}^{-m/2}{\left(\det g^{slow}\right)}^{1/2}\exp\left(-\frac{1}{2}\sum_{\nu^{\prime },\nu^{\prime \prime }=1}^{m}a_{\nu^{\prime }}g_{\nu^{\prime }\nu^{\prime \prime }}^{slow}a_{\nu^{\prime \prime }}\right)$$

where $g^{slow}$ is now an m×m matrix.

There are two methods to find this reduced matrix. The first and more simple one is that the equilibrium correlations in the reduced description should be given by the right hand side of Equation (23) for the correlation function. This results in

(30) $${\left[{\left(g^{slow}\right)}^{-1}\right]}_{\nu \nu^{\prime }}={\left[g^{-1}\right]}_{\nu \nu^{\prime }}\;\;\;\mathrm{for}\;\;\;\nu ,\nu^{\prime }=1,...,m$$

The inverse of the reduced matrix is therefore equal to the m×m submatrix for the slow eigenvectors of the inverse of the original g matrix.

In order to verify that this result is consistent with the straightforward integration of Equation (27), I also verify Equation (30) via direct integration of Equation (27). Write

(31) $$P_{eq}^{slow}\left(A_{\nu };\nu =1,...,m\right)={\left(2\pi \right)}^{-n/2}{\left(\det g\right)}^{1/2}\int da(\prod_{\nu =1}^{m}\delta \left(A_{\nu }-a_{\nu }\right)\exp\left(-\frac{1}{2}a.g.a\right))$$

Next, use $\delta \left(x\right)=\frac{1}{2\pi }\int dk\exp\left(ikx\right)$ and write

(32) $$P_{eq}^{slow}\left(A_{\nu };\nu =1,...,m\right)={\left(2\pi \right)}^{-m-n/2}{\left(\det g\right)}^{1/2}\int d^{m}k\int da\exp\left(ik.\left(a-A\right)-\frac{1}{2}a.g.a\right)$$

where $k\equiv (k_{1},...,k_{m},0,...,0)$ and $A\equiv (A_{1},...,A_{m},0,...,0)$. The expression in the exponent may then be written as

$$ik.\left(a-A\right)-\frac{1}{2}a.g.a=-\frac{1}{2}\left(a-ig^{-1}.k\right).g.\left(a-ig^{-1}.k\right)+ik.A-\frac{1}{2}k.g^{-1}.k$$

Upon substitution into Equation (32), one may perform the integral over a, which is now a simple Gaussian integral, and one obtains

(33) $$P_{eq}^{slow}\left(A_{\nu };\nu =1,...,m\right)={\left(2\pi \right)}^{-m}\int d^{m}k\exp\left(ik.A-\frac{1}{2}k.g^{-1}.k\right)$$

The vectors k and A may now be reduced to their *m* dimensional form. Notice that the exponent now contains the m×m dimensional submatrix of $g^{-1}$. This m×m dimensional submatrix is by definition equal to ${\left(g^{slow}\right)}^{-1}$. Writing

$$ik.A-\frac{1}{2}k.{\left(g^{slow}\right)}^{-1}.k=-\frac{1}{2}\left(k-ig^{slow}.A\right).{\left(g^{slow}\right)}^{-1}.(k-ig^{slow}.A)-\frac{1}{2}A.g^{slow}.A$$

one obtains upon substitution into Equation (33) and integration over k

(34) $$P_{eq}^{slow}\left(A\right)={\left(2\pi \right)}^{-m/2}{\left(\det g^{slow}\right)}^{1/2}\exp\left(-\frac{1}{2}A.g^{slow}.A\right)$$

This is equivalent to Equation (29).

The importance of the analysis in the last § is that the equilibrium distribution of the slow variables in fact follows directly from the equilibrium ensemble. Whether one directly reduces the microscopic description to these slow variables or does it via an intermediate macroscopic description using a larger number of variables, some of which are fast compared to the final slow variables, this method leads to exactly the same “initial condition” of the correlation functions of the slow variables. This is an important step to check the internal consistency of the procedure.

## 5. The Langevin Equation for the Slow Amplitudes

Now, turn to the derivation of the Langevin equation for the slow variables

(35) $$\frac{d}{dt}a_{\nu }\left(t\right)=-\sum_{\nu^{\prime }=1}^{m}L_{\nu \nu^{\prime }}^{slow}a_{\nu^{\prime }}\left(t\right)+f_{\nu }^{slow}\left(t\right)\;\;\;\mathrm{for}\;\;\;\nu =1,...,m$$

The most convenient method to obtain this equation is to first consider the average of this equation

(36) $$\frac{d}{dt}\langle a_{\nu }\left(t\right)\rangle =-\sum_{\nu^{\prime }=1}^{m}L_{\nu \nu^{\prime }}^{slow}\langle a_{\nu^{\prime }}\left(t\right)\rangle \;\;\;\mathrm{for}\;\;\;\nu =1,...,m$$

where the fact that the average of the reduced random force is also zero is used

(37) $$\langle f_{\nu }^{slow}\left(t\right)\rangle =0$$

Using Equation (20), it follows that

(38) $$L_{\nu \nu^{\prime }}^{slow}=e_{\nu }^{l}.L.e_{\nu^{\prime }}^{r}=\lambda_{\nu }\delta_{\nu \nu^{\prime }}\;\;\;\mathrm{for}\;\;\;\nu ,\nu^{\prime }=1,...,m$$

It then follows that

(39) $$\langle f_{\nu }^{slow}\left(t\right)f_{\nu^{\prime }}^{slow}\left(t^{\prime }\right)\rangle =2{𝓁}_{\nu \nu^{\prime }}^{slow}\delta \left(t-t^{\prime }\right)$$

with

(40) $$L_{\nu \nu^{\prime }}^{slow}=\sum_{\nu^{\prime \prime }=1}^{m}{𝓁}_{\nu \nu^{\prime \prime }}^{slow}g_{\nu^{\prime \prime }\nu^{\prime }}^{slow}\;\;\;\mathrm{or}\;\;\;{𝓁}_{\nu \nu^{\prime }}^{slow}=\sum_{\nu^{\prime \prime }=1}^{m}L_{\nu \nu^{\prime \prime }}^{slow}{\left[{\left(g^{slow}\right)}^{-1}\right]}_{\nu^{\prime \prime }\nu^{\prime }}$$

With Equations (30) and (38), the last equality reduces to *m* of the original variables. Choose for this purpose a subset which remains independent for t≫τ. Relabel the original variables such that this subset consists of the first *m* variables. Define

(41) $${𝓁}_{\nu \nu^{\prime }}^{slow}=\sum_{\nu^{\prime \prime }=1}^{m}e_{\nu }^{l}.L.e_{\nu^{\prime \prime }}^{r}e_{\nu^{\prime \prime }}^{l}.g^{-1}.e_{\nu^{\prime }}^{r}=\lambda_{\nu }e_{\nu }^{l}.g^{-1}.e_{\nu^{\prime }}^{r}$$

In view of the fact that the matrix g^−1^ is symmetric, it follows that the reduced Onsager matrix in Equation (41) is symmetric.

## 6. A Reduced Description Using the Original Variables

An aspect of the use of the slow amplitudes is that they may be unpleasant combinations of the original variables. One may alternatively use a subset

(42) $$a^{red}\left(t\right)\equiv \left(a_{1}\left(t\right),...,a_{m}\left(t\right)\right)$$

When t≫τ, this reduced set of variables is a linear combination of the amplitudes of the slow eigenfunctions. This implies that the use of the reduced set corresponds to the choice of a new basis set in the slow variable space. The Langevin equation is valid for all choices of the basis, and therefore also for $a^{red}$ using $L^{red}$, ${𝓁}^{red}$ and $g^{red}$.

## 7. Conclusions

After this long analysis, one may draw some conclusions:

(i) At any time scale, one has a natural choice of variables for which the relaxation from an initial state and the fluctuations are described in the usual way.

(ii) If one reduces a description by elimination of fast variables, both the linear (Onsager) coefficients and the equilibrium distribution change their values.

(iii) The reduced Onsager matrix ${𝓁}^{red}$ and the correlation matrix $g^{red}$ are not trivially obtained from *ℓ* and g. For this, one needs the eigenvectors of L=𝓁.g.

(iv) The Onsager matrix ${𝓁}^{red}$ for the slow variables and the correlation matrix $g^{red}$ are symmetric.

(v) Except for the case where one can conduct experiments on the fast and the slow time scale, there is no need to carry out this reduction. One simply uses a set of independent variables which have not relaxed and constructs $L^{red}$, ${𝓁}^{red}$ and $g^{red}$ on the basis of physical arguments alone.

(vi) The initial state, equilibrium distribution and entropy depend on the time scale one considers. On each time scale, it will be such that processes that decay on a faster time scale have equilibrated when the experiment or simulation is started.

(vii) The initial state, equilibrium distribution and entropy for the reduced description cannot be found by substituting the equilibrium values of the fast variables in the more extended description.

To sum up, I have shown that the reduction in slow variables does not affect the structure of the equations we use. It does not affect the validity of the Onsager relations and the symmetry of the correlation functions.

Finally, notice that a coordinate transformation does not change the eigenvectors and eigenvalues of a matrix. Therefore, $L^{red}$ and $L^{slow}$ have the same eigenvectors and eigenvalues. Accordingly, one finds that all the relaxation times remain the same.

## Data Availability

Data are contained within the article.

## References

[1] Onsager L. (1931). Reciprocal relations in irreversible processes. I. Phys. Rev..

[2] Onsager L. (1931). Reciprocal relations in irreversible processes. II. Phys. Rev..

[3] Casimir H.B.G. (1945). On Onsager’s principle of microscopic reversibility. Rev. Mod. Phys..

[4] Onsager L., Machlup S. (1953). Fluctuations and irreversible processes. Phys. Rev..

[5] Callen H.B., Welton T.A. (1951). Irreversibility and generalized noise. Phys. Rev..

[6] Green M.S. (1954). Markoff random processes and the statistical mechanics of time-dependent phenomena. II. Irreversible processes in fluids. J. Chem. Phys..

[7] Kubo R. (1966). The fluctuation-dissipation theorem. Rep. Prog. Phys..

[8] Geigenmüller U., Titulaer U.M., Felderhof B.U. (1983). Systematic elimination of fast variables in linear systems. Phys. A Stat. Mech. Appl..

[9] van Kampen N.G. (1985). Elimination of fast variables. Phys. Rep..

[10] Hubmer G.F., Titulaer U.M. (1987). The Onsager-Casimir relations revisited. J. Stat. Phys..

[11] Titulaer U.M. (1988). On the microscopic background of the Onsager-Casimir reciprocity relations. J. Stat. Phys..

[12] Cimmelli V.A., Sellitto A., Jou D. (2014). A nonlinear thermodynamic model for a breakdown of the Onsager symmetry and the efficiency of thermoelectric conversion in nanowires. Proc. Roy. Soc..

[13] de Groot S.R., Mazur P. (1962). Nonequilibrium Thermodynamics.

[14] Hynes J.T. (1972). On Hydrodynamic Models for Brownian Motion. J. Chem. Phys..

[15] Burgess R.E. (1973). Brownian motion and the equipartition theorem. Phys. Lett. A.

[16] Widom A. (1971). Velocity fluctuations of a hard-core Brownian particle. Phys. Rev. A.

[17] Mazo R.M. (1971). Theory of Brownian motion. IV. A hydrodynamic model for the friction factor. J. Chem. Phys..

[18] Chow T.S., Hermans J.J. (1974). Limitations of the Langevin equation based on hydrodynamic models. Proc. Koninkl. Nederl. Akad. Wet. Ser. B.

